# Comprehensive Algorithm for Nasal Ala Reconstruction: Utility of the Auricular Composite Graft

**DOI:** 10.1055/s-0038-1639581

**Published:** 2018-04-18

**Authors:** Collin Chen, Ruchin Patel, John Chi

**Affiliations:** 1 Division of Facial Plastic and Reconstructive Surgery, Department of Otolaryngology- Head and Neck Surgery, Washington University in Saint Louis School of Medicine, St. Louis, Missouri

**Keywords:** nasal ala, nasal reconstruction, composite graft, locoregional flap, facial reconstruction

## Abstract

Defects of the nasal ala are challenging to reconstruct, given its complex three-dimensional structure. Successful repair of these defects needs to provide aesthetic symmetry and preserve nasal function. A wide variety of reconstructive options have been described for nasal ala defects, ranging from skin grafts to locoregional flaps, and also includes the auricular composite graft. However, there are currently no comprehensive guidelines for nasal ala repair, and the versatile role of the auricular composite graft has not been well defined. In this review, we aim to provide a comprehensive algorithm to guide repair of nasal ala defects. Additionally, we compare our experience using the auricular composite graft with the available literature to better define its utility in nasal ala repair.

The nasal ala is a paired structural subunit of the nose that is vital for maintaining nasal symmetry and functionally important in the maintenance of the nasal valve. For skin cancers of the ala, surgical resection is usually the first-line treatment. The resulting defects at this unique location are often ill-suited for healing by secondary intention, and typically cannot be closed primarily. Furthermore, the nasal ala creates natural creases with the cheek, nasal sidewall, and nasal tip that need to be maintained. Thus, repair of nasal ala defects can be a challenging operation for facial reconstructive surgeons.

The nasal ala consists of three anatomically distinct layers—the external skin, the internal nasal lining, and the fibrofatty middle portion. Typically, this middle portion needs to be reconstructed with a nonanatomic graft as this unique tissue cannot be replaced. Each layer is important in the reconstruction to integrate seamlessly with the nose, prevent scar contracture, and maintain nasal patency. 1 2 When multiple layers are involved in a defect, the reconstruction requires combining procedures to replace each layer, such as an interpolated cheek flap combined with a free cartilage graft. 3 This results in multiple surgical donor sites and can require staged surgeries to accomplish an acceptable outcome.

The technique of using autologous skin with underlying auricular conchal cartilage (composite graft) for nasal reconstruction was first introduced by Konig in 1902. 4 Since its description, the auricular composite graft has been used in nasal reconstruction for a variety of purposes. It has been proven effective in vestibular stenosis repair, nasal valve insufficiency, external skin and cartilage reconstruction, and intranasal lining repair in full thickness nasal defects. 5 6 7 8 Auricular composite grafting is an attractive option for nasal ala reconstruction given that the conchal skin is a good color match and the conchal cartilage mimics the natural contour of the ala. 9 Furthermore, the surgical technique can be performed quickly with relative ease and leaves little donor site morbidity for the patient. 2 10

There is extensive literature regarding the reconstructive options and their outcomes for the external nasal skin, ranging from skin grafts to locoregional flaps; 3 11 however, defects at the nasal ala are unique in their impact on aesthetic outcome and nasal function. They require different surgical approaches than other nasal subunits. Currently, there are no comprehensive guidelines available that outline all available options for each layer of the ala. The goal of this review is to provide a detailed algorithm for partial and full-thickness nasal ala reconstruction. In addition, we will describe our outcomes using the auricular composite graft, and help define its role in this reconstructive algorithm.

## Algorithm

Prior to developing an algorithm for nasal ala reconstruction, the alar defect must first be categorized. We believe the most informative way to classify alar defects is based on thickness and size. By definition, all defects will involve the alar skin, and this represents the most superficial category of thickness. If the alar defect includes much of the fibrous middle layer, the surgeon's reconstructive options must consider harvesting cartilage. Finally, if the defect is full thickness, the surgeon must reconstruct all three layers of the ala. Thus, defining the defect based on thickness is closely related to the number of elements necessary to repair the wound. Within each category of thickness, the size of the defect dictates the area of each reconstructive element needed to repair the wound. Other aspects of the defect the surgeon must consider include skin color, skin laxity, and amount of adjacent skin available for recruitment. We acknowledge that gauging size guidelines for repairing soft tissue defects and assessing aesthetic outcomes can be a subjective process. These assessments are open to surgeon and patient interpretation, and we encourage all options to be considered when deciding repair. As such, we advise that surgeons consider these sizing guidelines as suggested values, rather than hardline mandates.

## Skin Only

**Fig. 1:**
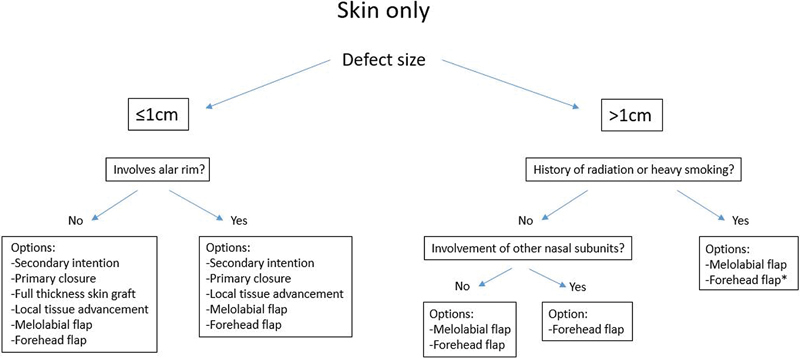
Options for reconstruction of superficial depth alar defects. *Denotes the recommended option.

If the nasal ala defect is limited to the skin and the lower lateral cartilage is intact, reconstruction can proceed without consideration for providing structural support to the ala ( Fig. 1 ). Alternatively, for deeper defects involving cartilage and nasal lining, the same principles for reconstructing the skin layer will still be applied to those reconstruction algorithms. Next, the surface area of the defect must be measured. Defects less than or equal to 1 cm are good candidates for full-thickness skin grafting. Skin defects larger than 1 cm occupy a significant percentage of the nasal ala subunit, and are thus more suited for local soft tissue flaps capable of replacing the subunit, such as the melolabial flap and the forehead flap. 12 13 Additionally, if the inferior limit of the skin defect involves the anterior alar rim, skin grafting should be used with great caution as it can result in alar notching. 13 In regard to local flaps for skin defects, we caution against using the bilobed flap and dorsal nasal flap for alar reconstruction due to their limited reach. For these flaps, the tissue recruited is usually superior or lateral of the level of the ala, which can lead to alar notching following retraction of the soft tissue. Furthermore, these flaps have the potential to blunt the alar groove, an important aesthetic landmark, which can be extremely challenging to fix secondarily. 13 14

*Full thickness skin graft* –Skin grafts are free grafts which have been completely separated from their blood supply. As such, a prerequisite for skin grafting is a wound bed that is capable of providing vascularity to the free skin graft. Avascular surfaces such as bone and cartilage are incapable of supporting a skin graft. 15 Unlike skin defects of the nasal sidewall and dorsum, which has the potential to expose high surface areas of upper lateral cartilage and nasal bone, skin defects of the nasal ala only has the potential to expose the lower lateral cartilage. When this occurs, there is usually well-vascularized subcutaneous tissue or perichondrium that is exposed along with the lower lateral cartilage. Perhaps the greatest challenge in achieving a good outcome with skin graft is color match. Potential candidates include skin from the melolabial fold, forehead, and preauricular skin. McCluskey et al advocates use of forehead skin due to similar degree of sun exposure and matching sebaceous character to the alar skin. 13

*Melolabial flap* –The melolabial flap is a pedicled transposition soft tissue flap which can be advanced medially toward the nasal ala to repair superficial and full-thickness defects. Its blood supply is derived from a random network of vessels from the distal facial and angular arteries. The surgeon has the option of utilizing this flap as either a single-stage or two-stage procedure for alar reconstruction. Both techniques involve raising the flap from the ipsilateral melolabial cheek, with the deepest part of the crease marking the inferior aspect of the donor flap so that it may be transposed toward the ala with a superiorly based pedicle. Burget and Menick have initially encouraged elevating a flap 1 mm larger than the alar defect. 16 More recent technique updates by Thornton et al have advocated for elevating a flap that is the same size, or slightly smaller, so that inset at the ala can be under tension to prevent pin-cushioning. 17 Fat on the distal end of the flap is thinned aggressively to prevent trap-door deformity. For one-stage reconstruction, during inset of the flap, two nylon sutures can be placed from the deep side of the flap into the nasal vestibular skin permanently to recreate the alar crease. Lindsey's series of 105 cases of single-stage melolabial flap reconstruction of superficial to full-thickness alar defects resulted in no complete flap failures, with seven cases of partial necrosis at the distal end of the flap. 18 In contrast, in their review of alar defects reconstructed with melolabial flaps, Driscoll and Baker cautions that single-stage use generally results in more revision procedures to reconstruct the alar-facial sulcus. 3 If the flap is inset as a two-stage procedure, the pedicle of the melolabial flap can be divided at 3 weeks during a second procedure, along with excision of the donor bulge and closure of the superior melolabial fold.

**Fig. 2:**
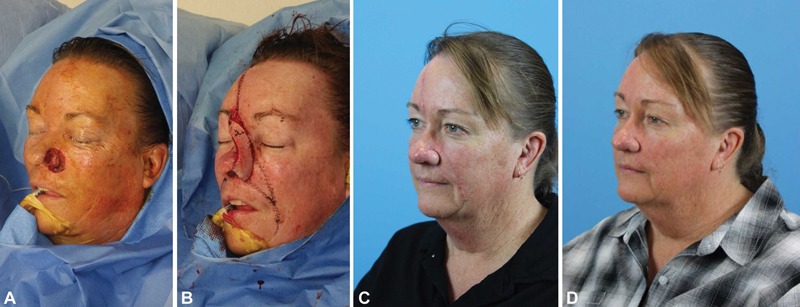
( **A** ) A patient with a large full-thickness defect of the ala which involves additional nasal subunits. ( **B** ) Reconstructed with a right forehead flap, island cheek advancement flap, inferior turbinate flap, and free auricular conchal cartilage graft. Appearance at ( **C** ) 2 months and ( **D** ) 4 months after surgery.

*Forehead flap* –The paramedian forehead flap (PMFF) is an interpolated flap based on an axial blood supply from the supratrochlear artery and perforators from the supraorbital artery. This reliable axial blood supply has established the forehead flap as the gold standard for nasal subunit reconstruction. Radiation therapy and smoking are factors that have been shown to increase risk of complications and flap compromise following melolabial flap. 19 For these patients, melolabial flaps should be used cautiously, and elevating the flap with a wider pedicle of 2 cm will improve flap survival. Otherwise, the robust axial blood supply of the forehead flap can provide a more reliable reconstructive option. 19 20 Additionally, the PMFF is reliable for patients of all ages and can be performed under anticoagulation. 21 The flap is elevated from the contralateral forehead to allow medial rotation at a relaxed pivot point. The PMFF is capable of replacing the entire alar subunit ( Fig. 2 ). If this is the case, the contralateral ala can be used as a template to guide inset and sculpting of the distal flap. Because the forehead flap is an interpolated flap, it is a two-stage procedure without an option for single-stage modification. At 4 weeks, division and inset can occur following a reliable period of vascularization. During division and inset, the flap can be further thinned, although this should be done cautiously in smokers. In addition, the skin of the ala can be elevated as much as 70% to allow further sculpting and placement of contouring sutures. 21

## Cartilage/Fibrous Layer

**Fig. 3:**
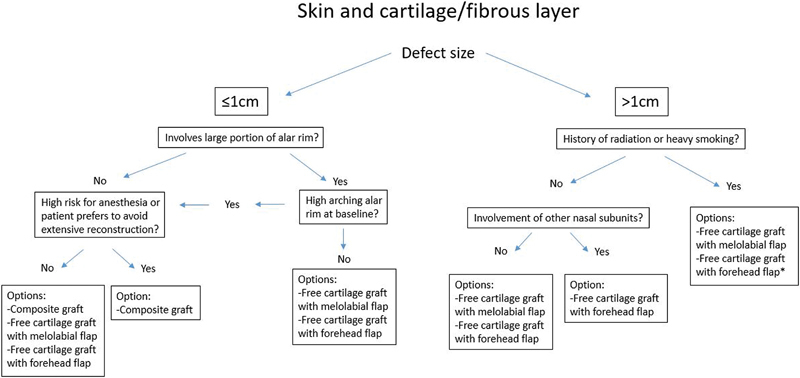
Options for reconstruction of intermediate depth alar defects. *Denotes the recommended option.

For nasal ala reconstruction, cartilage grafting can be used to restore the natural convexity to the ala as well as provide support to the alar rim to prevent notching and external nasal valve collapse ( Fig. 3 ). In certain cases when the lower lateral cartilage remains anatomically intact in a defect, a cartilage graft may still be needed to provide aesthetic support to the ala. This is especially true if a bulky soft tissue flap is planned, such as a melolabial flap or forehead flap. Similarly, although the alar rim contains no anatomic cartilage, defects involving the rim may require nonanatomic grafting to prevent delayed complications such as alar notching. 17 This is especially true if the defect involves a large portion of the alar rim, such as when the widest portion of the defect occurs at the alar rim. If support to the ala is not established during primary reconstruction, the soft tissue envelope may collapse following scar contracture, making it difficult to regain the proper support needed to prevent nasal airway obstruction. 14

*Free cartilage graft* –In nasal reconstruction, cartilage grafts are most frequently harvested from the nasal septum and auricle. For alar reconstruction, although septal cartilage can be used to construct the alar framework, auricular cartilage is preferred. The natural curvature of the conchal cartilage is well suited for recreating the convexity of the ala. The cartilage from the concha cymba and concha cavum can be approached from an anterior or posterior skin incision. Once harvested, the conchal cartilage can be shaped and trimmed to match the native alar curvature, and can be thinned down to 1 mm while maintaining its convexity. During placement of the graft, a small pocket should be dissected at the alar facial sulcus to anchor the lateral aspect of the graft to prevent prolapse into the vestibule. Driscoll and Baker reviewed 50 patients with alar defects requiring free cartilage grafts and concluded that it produces good aesthetic results when combined with additional reconstruction procedures. They report a partial loss of skin flap in 6% of the patients when combined with a forehead flap or melolabial flap, and partial loss of lining flap in 8% of the patients when combined with intranasal lining repair. 3 Free cartilage grafting is surgically reliable, can be easily combined with other locoregional flaps in alar reconstruction, and has been established as the gold standard for skeletal support in intermediate and full-thickness alar defect reconstruction.

*Composite cartilage graft* –Auricular cartilage composite graft is a free tissue graft involving the conchal cartilage and overlying skin. This one-stage procedure has the potential to repair skin and cartilage defects during one surgery from one donor site. Like skin grafts, the composite graft relies on the wound bed to provide vascularization to support the tissue. Unlike skin grafts, the composite graft is much thicker, and only small composite grafts can be sustained purely on imbibition of nutrients from the wound bed. 2 8 22 23 The composite graft has three potential uses in alar reconstruction. First, for intermediate depth defects where the internal lining is present, it can provide cartilage support and external skin coverage. In this case, the composite graft is harvested from the posterior concha so the skin side matches the convexity of the alar skin. Second, for full-thickness defects, the auricular skin can replace the internal lining while an additional vascularized flap, either a melolabial flap or forehead flap, is used to sustain the graft and provide external skin coverage. In this case, the composite graft is harvested from the anterior concha so the skin side matches the concavity of the lining. Third, for full-thickness defects, both the anterior and posterior conchal skin can be harvested and transferred as a three-layer graft.

The survival of the composite graft is dependent on multiple factors, but the most important limitation is size. 24 Teltzrow et al analyzed outcomes of composite grafting for two-layer reconstruction of the external nasal skin, and were able to achieve low rates of complication for grafts ≥ 2 cm when gentle scarification and heparin treatments were applied postoperatively. 22 In their cohort of 91 patients receiving composite grafts for reconstruction of the inner lining, Scheithauer et al found no graft necrosis for grafts ≤ 2 cm, and suggests this is the critical cutoff size. 2 However, this cohort not only includes patients receiving composite grafts with external locoregional flaps for full-thickness defects, but also includes patients with no external skin defects, and defects not located at the ala. Thus, the true outcomes for composite grafting to isolated alar defects are unclear. Comparatively, Haas and Glogau suggests that for full-thickness defects, composite grafts larger than 1.5 cm have failure rates > 50%. 25 Other technical modifications aimed to improve graft survival are designed to increase the contact surface between the graft and tissue bed to maximize vascularization. Avoiding thermocoagulation during surgery, strong counseling against smoking, and protection from shearing trauma postoperatively will also help maintain adequate vascularization to the graft and improve survival. 23 26 27 28 Harbison et al reviewed the literature on auricular composite grafting for nasal reconstruction and concluded that interventions which may improve survival include perioperative steroid therapy, applying cooling packs to the graft postoperatively, and consideration for hyperbaric oxygen therapy in difficult cases. 24 However, without technical and postoperative modifications, auricular composite grafting is likely to be reliable only for defects in the range of 1 to 1.5 cm based on our review of the available literature. 14 24 29 30

**Fig. 4:**
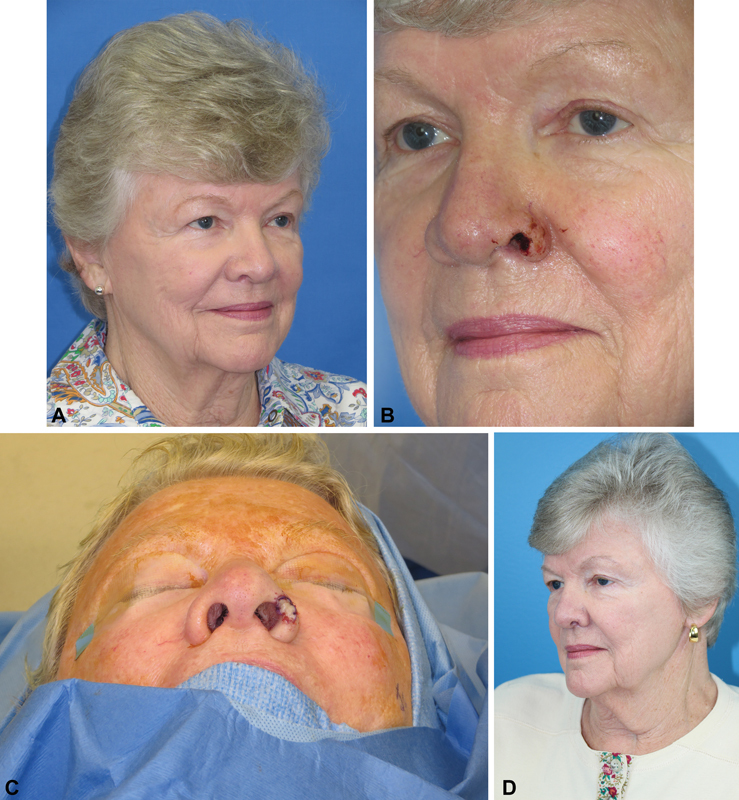
( **A** ) A female with high arching alar rim at baseline with ( **B** ) a 1-cm full-thickness defect of the ala involving the rim. ( **C** ) Auricular composite graft used to reconstruct the defect with ( **D** ) good aesthetic result at 2 months after surgery.

In our experience using composite grafts for partial and full-thickness ala defects, the best results occurred when the graft was 1 cm or less. Very few patients in our series with defects larger than 2 cm had good aesthetic outcomes. Thus, we advocate the more conservative cutoff of 1 cm as the upper limit of alar defects suitable for composite grafting. We made sure to support the composite graft adequately by advancing external skin as well as vestibular lining when appropriate. We reiterate that our suggestion of using the more conservative cutoff of 1 cm is a relative guideline based on the best results we observed. This cutoff is designed to be a recommendation rather than an exclusion criterion, and certainly does not preclude good outcomes beyond the size guideline. Complications we encountered with larger composite grafts included alar notching, external nasal valve collapse, hypertrophic scarring, and contour asymmetry. These patients required multiple secondary procedures such as steroid injections and laser treatments. Given these experiences, we recommend performing traditional auricular cartilage grafting with concurrent skin coverage through a locoregional flap for larger alar defects. Similar to skin grafting, composite grafting for small defects at the alar rim can potentially cause alar notching. But unlike skin grafts, the cartilage within the composite graft provides shape and structural support to the alar rim which can resist some degree of retraction. Patients who have high arching alar rims at baseline may be good candidates for composite grafting because slight alar notching would not create significant asymmetry ( Fig. 4 ). Additionally, it is worth considering harvesting an auricular composite graft from the helical root for cases where there is exact contour match with the alar rim defect. For smaller alar defects in patients with medical comorbidities who cannot tolerate prolonged general anesthesia, such as American Society of Anesthesiologists (ASA) class 4 or 5, the composite graft is a viable alternative with potential to drastically cut down on operating time. Furthermore, it has the advantage of avoiding a second surgery for division and inset, which is difficult to bypass when reconstructing with melolabial and forehead flaps. Thus, the composite graft would also be a viable alternative if the patient does not want an extensive reconstruction with a two-stage procedure.

## Lining

**Fig. 5:**
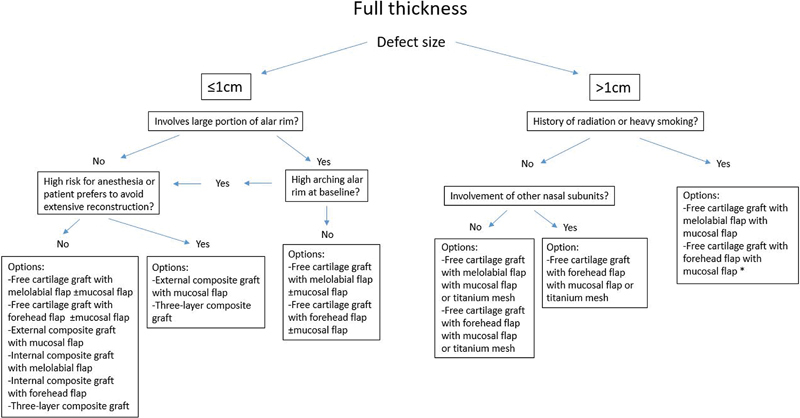
Options for reconstruction of full thickness alar defects. *Denotes the recommended option.

In reconstructing a full-thickness alar defect, one must incorporate lining repair into the surgical algorithm ( Fig. 5 ). A good option is a bipedicled vestibular advancement flap, based medially at the junction of the anterior vestibular lining with the septum and laterally at the nasal floor. After making a superior incision in the vestibular lining, this bipedicled flap can be elevated and rotated inferiorly and carefully sewn into the inferior wound edge of the defect. A skin graft may be necessary to replace the donor site to prevent scar contracture leading to alar notching. Due to the delicate nature of this dissection, the vestibular advancement flap can provide coverage for roughly 1 cm of nasal lining. 1 31 For larger lining defects, more involved pedicled mucosal flaps can be used. The inferior turbinate can be elevated based on an anterior pedicle from branches of the angular artery and turned toward the ala. After removal of the turbinate bone, the mucosa can be peeled open to yield a flap of 1.7 cm in width and 2.8 cm in length. Further reach can be gained by elevating the nasal floor and inferior meatal mucosa in continuity with the turbinate. This flap does have the disadvantage of potentially needing a secondary surgery for pedicle division. 1 32 Another option is an ipsilateral septal mucosal flap based medially and anteriorly on the septal branch of the superior labial artery. This flap of mucoperichondrium is elevated posteriorly to anteriorly along the septum before making superior and inferior cuts to allow rotation of the flap laterally and anteriorly toward the ala. Using this flap will require a secondary surgery for pedicle division to relieve nasal airway obstruction. Additionally, there is donor-site morbidity related to crusting which may worsen nasal airway obstruction. 1 17 Surgeons must also keep in mind the possibility of combining different intranasal mucosal flaps to meet defect requirements.

For nonradiated patients, internal lining defects may also be repaired with titanium mesh. Titanium mesh is a structural implant strong enough to resist cicatricial contracture while permitting internal mucosalization. Zenga et al found that for patients with full-thickness nasal defects treated with a vascularized skin flap covering a molded titanium mesh, there was excellent implant incorporation and remucosalization. The internal surface granulated through the titanium mesh and no dedicated lining repair was necessary. This technique is mostly used for defects of the upper two-thirds of the nose, but can be applied to mucosal defects extending inferiorly into the ala. For full-thickness defects in nonradiated patients with a large mucosal deficit involving the ala and other subunits, repair with a melolabial flap or forehead flap combined with titanium mesh may be a good option. 1 33

## Conclusion

The nasal ala is functionally important for the nasal airway and is aesthetically important for nasal symmetry. Defects of the nasal ala are challenging to reconstruct, and each anatomic layer must be addressed during repair. Our algorithm outlines options for reconstruction based on the depth of the defect and size of the defect. The auricular composite graft is a versatile and valuable option that has multiple uses in nasal alar reconstruction. It can provide one-stage reconstruction and decrease operating time, but because graft survival is limited by size, it should be used judiciously and reserved for small alar defects. Adjunct therapy such as steroid therapy, resurfacing, and cooling may be necessary to improve graft survival. Regardless of the reconstruction method, patients should be counseled that close follow-up for postoperative interventions may be necessary to achieve the best aesthetic outcome.
